# Predictive bite force modelling of head and neck oncology patients for clinical mandibular reconstruction applications

**DOI:** 10.1007/s00784-025-06626-5

**Published:** 2025-10-28

**Authors:** Jorn-Ids Heins, Barzi Gareb, Reinier ten Brink, Nathalie Vosselman, Gyorgy B. Halmos, Anastasiia O. Krushynska, Bram B. J. Merema, Joep Kraeima, Max J. H. Witjes

**Affiliations:** 1 Department of Oral and Maxillofacial Surgery, University of Groningen, University Medical Center Groningen, Groningen, 9713 GZ The Netherlands; 2 3D Lab, University of Groningen, University Medical Center Groningen, Groningen, 9713 GZ The Netherlands; 3 Department of Special Dentistry, University of Groningen, University Medical Center, Groningen, Groningen, 9713 GZ The Netherlands; 4 Department of Otorhinolaryngology, Head and Neck Surgery, University of Groningen, University Medical Center Groningen, Groningen, 9713 GZ The Netherlands; 5 Engineering and Technology Institute Groningen, University of Groningen, Groningen, 9747AG The Netherlands

**Keywords:** Bite force, Masseter muscle, Magnetic resonance imaging, Linear models, Muscles, Mandibular reconstruction

## Abstract

**Objective:**

Since the introduction of individualized planning using 3D technology, the necessity for an individualized predictive bite force model has become increasingly critical for optimizing functional rehabilitation, and for tailoring surgical interventions to each head and neck oncology patient’s unique anatomical and biomechanical profile. Current predictive models often lack comprehensive predictors and robust statistical methodologies, limiting their clinical applicability. To overcome these challenges, a predictive model of head and neck oncology patients’ maximum voluntary bite force was developed by analyzing key anatomical and physiological factors.

**Results:**

Data were collected from 41 head and neck cancer patients, with 109 bite force observations analyzed using a linear mixed-effects model. The identified key predictors include body weight, superficial masseter muscle volume, bite force measurement region, and maxilla and/or mandibular teeth mobility. Superficial masseter muscle volume showed a positive association with bite force, while increased mobility of the teeth in the maxilla or mandible impacted bite force negatively.

**Conclusion:**

The model demonstrated strong explanatory performance (conditional R² = 0.777).

**Clinical relevance:**

This model enables patient-specific treatment planning and implant design for head and neck reconstructive surgery by implementing the bite forces in a finite element software used for computer aided designs. For instance, by tailoring mandibular reconstruction plates to individual bite forces using novel materials or designs, the model allows for a balance between sufficiently strong designs while preventing stress shielding seen with excessively strong designs. This study provides valuable insights into the multifactorial nature of bite force and its implications for patient care.

## Introduction

Bite force represents one of the most fundamental factors for evaluating masticatory function in patients undergoing oral and maxillofacial surgery, including maxillofacial trauma [1, 2], orthognathic corrections [3] and mandibular reconstruction [4, 5] patients. Three-dimensional (3D) technology allows for the design of patient-specific reconstruction or osteosynthesis plates. Although these plates are labelled as ‘patient-specific’ or ‘custom’, they are generally designed according to the shape of the jaw within fixed design parameters thus, ‘shape-specific’. This limits the design of patient-specific characteristics, and generally results in rigid reconstruction plates. Mandibular reconstruction plates that are too rigid lead to complications related to stress-shielding [6–8]. Nowadays, novel mandibular reconstruction plate designs utilize porous metamaterial structures that can be tailored to patient-specific bite forces, to reduce complications related to stress-shielding [9]. To enable the clinical implementation of such novel alternative plate designs [9], or novel materials [10], adequate patient-specific bite force predictions are necessary.

The magnitude of an individual bite force varies based on different parameters such as age, dentition, sex, site of measurement, occlusal characteristics, and (transient) tooth pain [11–17]. Limited models are available in the literature to predict bite force. Jabr et al. were able to explain 70% of the bite force variance among woman by using handgrip force [18]. Varga et al. explained 31.3% of the bite force variance demonstrated by subjects aged 15–18 years with normal occlusion through sex, age, number of occlusal contacts and body mass index (BMI) [19]. Other studies have associated bite force with other factors, including masseter thickness measured by ultrasound, and swallowing difficulty [20], demographic data and occlusion [21], and skeletal patterns [22]. However, they did not present a predictive model with quantified accuracy. Other models estimate mammalian bite force using body mass, skull width and phylogenetic relationships [23], and 3D skull geometry and physiological cross-sectional areas [24]. Although valuable, these models for predicting patient-specific bite force often fail to account for a complete array of predictors. Currently, there is no model for predicting bite force with sufficient explanatory power for head and neck oncology patients, which can be used for subsequent reconstruction plate designs. In this study, we aim to develop a comprehensive model for predicting maximum voluntary bite forces in head and neck oncology patients, by addressing the limitations of existing models.

## Materials and methods

### Study design

The study was conducted in the department of Oral and Maxillofacial Surgery and the Special Dental Care Center of the University Medical Center Groningen (UMCG), Groningen, the Netherlands. Between June 2024 and April 2025, patients suspected of having or being diagnosed with head- and neck cancer were included in this study. The study design was approved by the medical ethics review committee on February 16, 2024 (METc number: METc 2024/107). Data was stored and processed in accordance with UMCG guidelines. The medical ethics approval also included the Investigational Medical Device Dossier (IMDD) concerning the in-house developed bite force sensor.

### Bite force sensor development

A custom bite force sensor was developed for this study, based on the FlexiForce Economical Load and Force Measurement (ELF) system (Tekscan, Norwood, the United States of America). This system works with a piezoresistive Flexiforce B201 (Tekscan, Norwood, United States of America) force sensor placed in a custom-made 3D printed polyamide 12 (PA12) holder. The sensor’s holder is designed so that biting forces can be transmitted perpendicularly to the sensor’s active area. The sensor and the bite force sensor holder were placed in a bite block cover, which acts as a sleeve and prevents sensor contamination. Custom-made attachments were put on top of the sleeve to allow for comfortable biting and to protect the bite force sensor holder and the patient’s teeth and/or prosthesis. When assembled, the bite force sensor had a thickness corresponding to a mouth opening of 17.5 mm, thus allowing for a maximum biting force [25, 26]. To accommodate different patients and measurement locations, the custom-made attachments were designed varying heights to ensure a consistent mouth opening of 17.5 mm.

### Calibration

The FlexiForce sensor was calibrated daily before use, following the manufacturer’s guidelines. A calibrated and ISO9001-2015 certified (AE-B3G-C3-100 kg-6B) load cell (AE Sensors, Dordrecht, the Netherlands) was assembled with a pressure bench. The load cell was used to calibrate the FlexiForce sensor. The error was logged and saved together with the calibration file. This was repeated each day the measurements were performed.

### Patient inclusion

Patients suspected or diagnosed with head and neck cancer by the Department of Ear, Nose and Throat (ENT), and the Department of Oral and Maxillofacial Surgery (OMFS) underwent a dental examination by an oral hygienist at the Center for Special Dentistry at the UMCG. In collaboration with a dentist, the oral hygienist assessed the status of the dentition in detail. This is routinely performed for each dentate head and neck oncology patient to assess if dental extractions are required before potential radiation therapy. Eligible patients were invited to participate in the study voluntarily. Any patient that initially consented, but was later found to be non-instructible during the measurements, was assessed as frail and excluded retrospectively. All the participants provided written informed consent and received a hard copy of the form.

### Measurement procedure

The bite force was measured using the bite force sensor after its daily calibration. The sensor was placed at an appropriate location in the mouth, determined according to the patient’s dental condition and tumor location. Measurement locations were recorded using teeth element numbers for the maxilla and mandible. Once the sensor was placed, the patient was instructed to close their mouth slowly until contact was made with the bite force sensor, allowing placement verification and correction if necessary. Thereafter, the patient was asked to bite on the sensor as hard as possible. During biting, the visual analogue scale (VAS) pain score was obtained along with the maximum bite force. This measure was repeated for multiple locations in the mouth if possible, with a maximum of four locations. Each measurement was validated by confirming that the patient had exerted maximum effort. There was at least 30 s between measurements to minimize any influence of fatigue [27]. Any VAS scores higher than one were excluded, since pain may have influenced the maximum bite force. Pain measurements were not included because pain is often a temporary and highly variable condition, such as dental pain from a cavity, which may resolve quickly and does not reflect a patient’s baseline functional capacity. The bite force measurements were categorized into cuspid/incisor, premolar, and molar regions. Following the bite force measurements, the patient performed a hand pinching test using a hand dynamometer to assess maximum pinching force in both hands. Lastly, the most recent weight and height were recorded and verified using the electronic patient file. Note that weight and height, the components of BMI, were stored as separate variables.

The muscle volumes of the temporalis, deep masseter, superficial masseter and the medial pterygoids were derived bilaterally, using a deep learning segmentation algorithm [28]. This segmentation was verified manually for each patient. The average volume and cross-sectional area of each muscle were calculated by averaging the left and right sides. The cross sectional area of the muscles at the C3 vertebrae level was segmented on the MRI and/or Computed Tomography (CT) scans, as a measure of potential sarcopenia, using a validated deep learning algorithm, and was verified manually.

During the dental examination, the oral hygienist recorded the presence and mobility of dental elements. If a tooth was present in the measured location in the maxilla or mandible, its mobility [29] was graded (no mobility, mobility degree 1, mobility degree 2, or mobility degree 3). If no teeth were present, the dental status was classified as either edentulous, prosthesis on mucosa, prosthesis fixed on dental implants, or a prosthesis fixed on remaining dental teeth.

Demographic information was collected about each patient and stored in the electronic patient file, including age at the time of measurement, tumor characteristics [30] (location, size, cT-, cN- and M-staging), amount of medicine, the number of remaining teeth in the mandible and in the maxilla based on the periodontal status, and the most recent orthopantomogram and additional periapical X-Rays.

### Statistical analysis

The distribution of the continuous data was checked visually and supplemented by the Shapiro-Wilk test. The means and Standard Deviations (SD) of the normally distributed continuous variables were calculated. Skewedly distributed data were presented as median and interquartile range (IQR). Nominal data were presented as numbers with corresponding percentages. Missing data were imputed using predictive mean matching (PMM) through multiple imputation by chained equations (MICE). Five imputed datasets were created, using 50 iterations to ensure convergence of the imputation process. Rubin’s Rule [31] was used to pool the imputed datasets, by combining parameter estimates and their variances to provide a final estimate and its standard error, thereby accounting for both within- and between-dataset variability.

The mixed-effect linear regression model was used with bite force as a dependent variable. A mixed effect model was applied to account for repeated measurements (i.e., within subjects). The mixed models were estimated using maximum likelihood estimation. The included random effects were the subjects. A backward selection procedure was utilized to iteratively remove variables that did not improve the model. Between model development, model improvement was tested using likelihood ratio tests.

The assumptions of the linear mixed-effect models were tested and verified. To evaluate multicollinearity among the predictor variables, variance inflation factors (VIFs) were computed. A VIF threshold of 10 was used to identify potential multicollinearity issues. The R-squared (R^2^) values were calculated from the resulting model using Nakagawa and Schielzeth’s approach [32]. In addition, the Mean Absolute Error (MAE) was calculated based on a 10-fold cross-validation.

A simulation-based post-hoc power analysis was performed for each individual fixed effect included in the linear mixed-effect model. In total, 1000 simulations were performed for each analysis to estimate the power of each fixed effect of the final model.

All analyses were performed in R [33], version 4.4.2, using the *lme4*,* lmerTest*,* simr*,* mice*,* dplyr*,* purrr*,* knitr*,* kableExtra*,* haven*,* performance*,* sjPlot*,* car*,* caret*,* ggplot2*,* gridExtra* and *grid* packages. A *p* < 0.05 was regarded as statistically significant.

## Results

### Bite force sensor development, calibration and measurement procedure

**Fig. 1:**
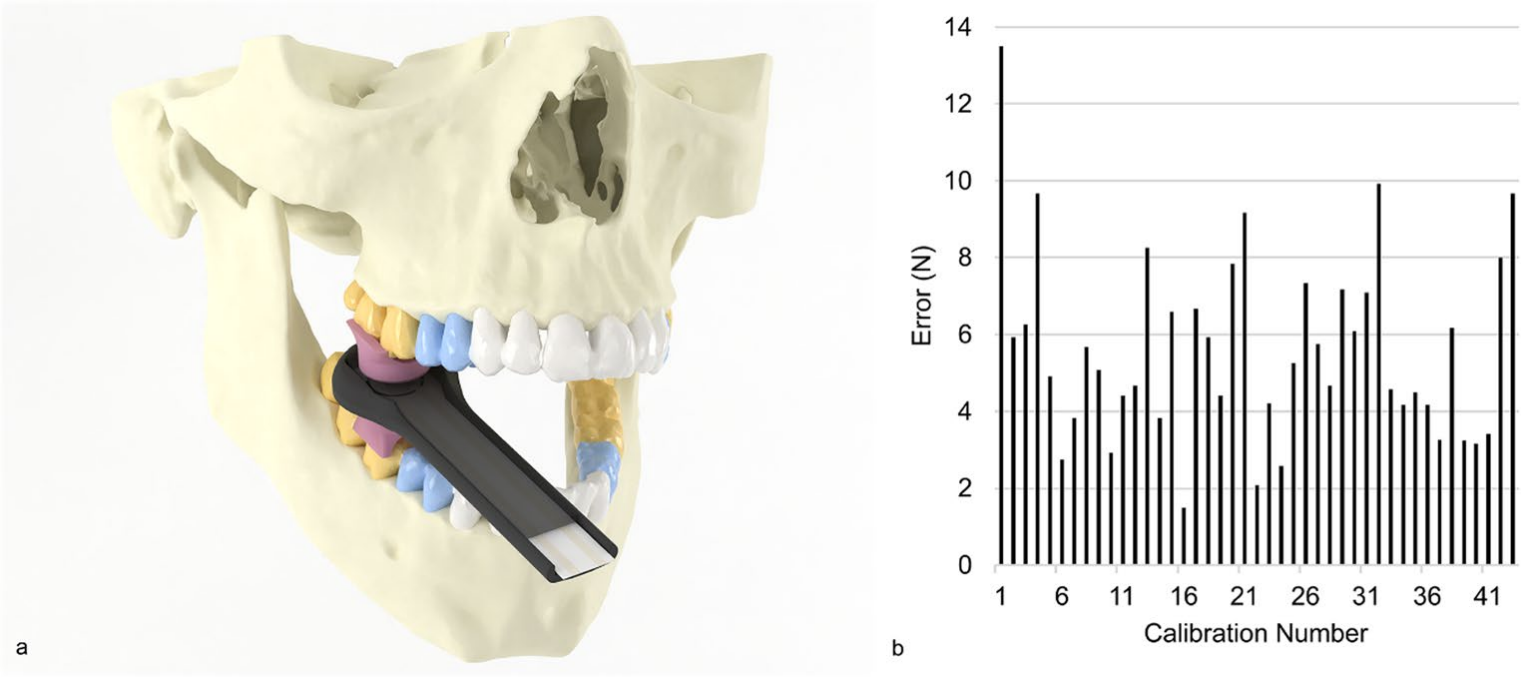
(**a**) bite Force Sensor. The Flexiforce B201 piezoresistive sensor is placed in custom 3D-printed PA12 holders. Customized soft attachments are placed on the holders. The holders allow for perfect perpendicular force alignment on the Flexiforce sensor. The different molar, premolar and cuspid/incisor measurement regions are colored yellow, blue and white, respectively. (**b**) Bite Force Sensor calibration errors. The maximum observed calibration error was 13.5 N, and the average error was 6 N

The developed bite force sensor was able to measure maximum point forces on multiple locations in the mouth. The developed bite force sensor, with the different measurement regions, is shown in Fig. 1a. The average error was equal to 5.6 N. The recorded errors are presented in Fig. 1b.

### Study population characteristics

**Fig. 2:**
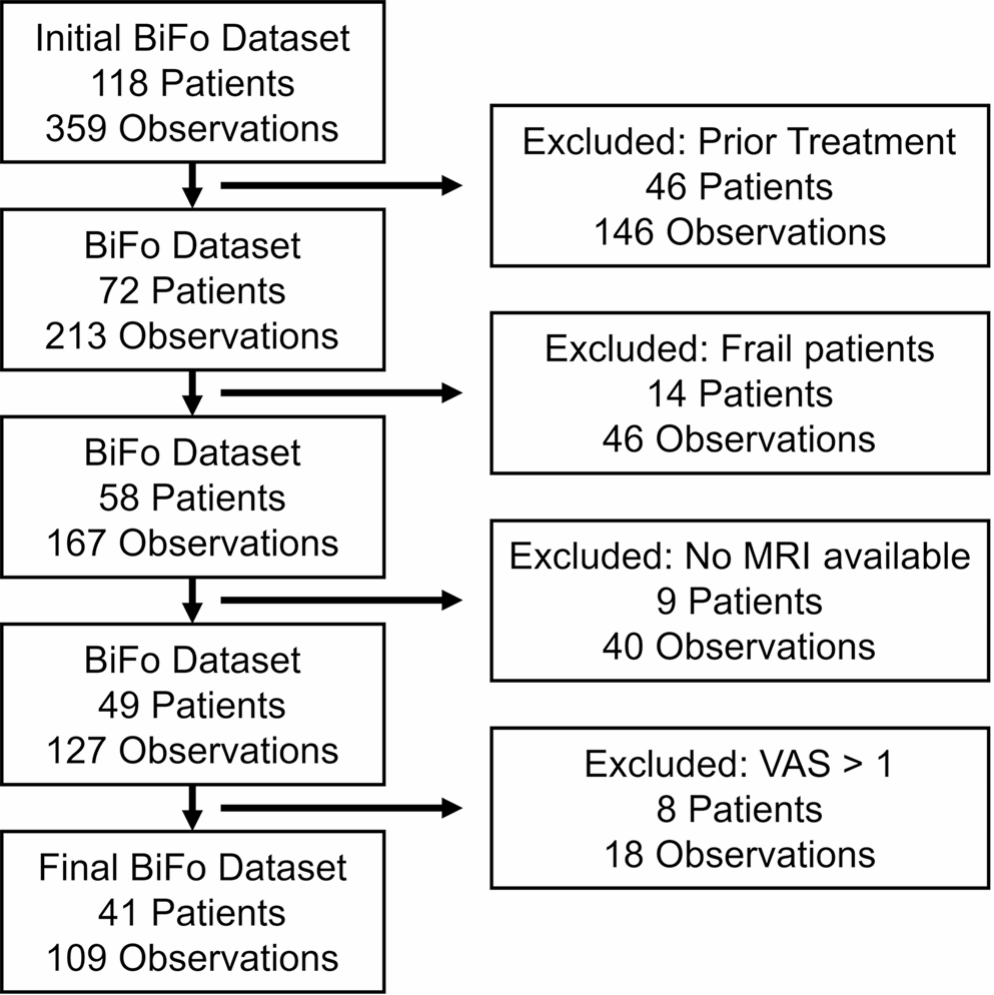
Flowchart illustrating the patient filtering process from the initial dataset (118 patients and 359 observations) to the final study population (41 patients and 109 observations). Sequential filters were applied to exclude patients based on prior treatment in the head and neck area, frailty status, availability of MRI scans, and VAS pain scores exceeding 1. Each filtering step reduced the number of patients and observations, resulting in a homogeneous cohort suitable for bite force prediction modelling

A total of 118 patients were initially recruited for the study but 77 patients had to be excluded, as shown in Fig. 2. Of those, 46 patients had undergone surgery or radiation therapy in the head and neck area, 14 patients were assessed as being frail (i.e. not instructible), 9 patients had not been MRI scanned, and 8 patients’ VAS assessment was higher than one, which could influence the maximum bite force measurements. Consequently, a total of 41 patients with 109 bite force measurements were included in the current study. Thus, only pain-free measurements performed prior to treatment at patients assessed as not frail are retained in the cohort. The baseline characteristics of the cohort are presented in Table 1.

**Table 1:** Baseline characteristics of the head and neck oncology patients included for subsequent analysis

Characteristic	Value
*Demographic characteristics*	
Age, years (mean [IQR] (range))	64.0 [59.0–73.0] (24–85)
Sex, n (%)	
Female	18 (43.9)
Male	23 (56.1)
Height, cm (mean ± SD (range))	174.8 ± 9.5 (160–195)
Weight, kg (mean ± SD (range))	78.9 ± 16.2 (43–109)
*Clinical characteristics*	
Polypharmacy, number of medications (mean ± SD (range))	6.2 ± 4.2 (0–16)
Tumor location, n (%)	
Not applicable	1 (2.4)
Oral cavity	18 (43.9)
Oropharynx	13 (31.7)
Hypopharynx	2 (4.9)
Parotid gland	3 (7.3)
Nasopharynx	4 (9.8)
Mobility degree teeth maxilla, n (%)	
Maxillary teeth no mobility	105 (96.3)
Maxillary teeth mobility 1	4 (3.7)
Mobility degree teeth mandible, n (%)	
Mandibular teeth no mobility	101 (92.7)
Mandibular teeth mobility 1	7 (6.4)
Mandibular teeth mobility 2	1 (0.9)
Teeth, n (median [IQR] (range))	25 [21–28] (6–30)
*Muscle characteristics*	
Muscle volumes, cm^3^ (mean ± SD (range))	
Temporalis	23.3 ± 6.9 (10.1–37.2)
Deep masseter	3.8 ± 1.0 (2.3–6.5)
Superficial masseter	14.3 ± 3.8 (8.0–21.8.0.8)
Medial Pterygoid	8.2 ± 2.0 (4.8–13.0)
Cross-sectional muscle area at C3 level, mm^2^ (median [IQR] (range))	37.2 [27.4–43.1] (22.1–52.3)
*Force measurements*	
Bite force, N (median [IQR] (range))	120 [80–160] (26–370)
Pinch force, N (median [IQR] (range))	38.0 [29.5–48.0] (21–77)

### Data preparation and preliminary analysis

Prior to estimating the linear mixed-effects model, multicollinearity was assessed by inspecting the VIF. Tumor location (VIF 23.1), Sex (VIF 10.0), Average Volume of the Temporalis Muscle (VIF 7.9) and the Average Volume of the Medial Pterygoid Muscle (VIF 6.1) showed high multicollinearity and, thus, were removed from the models.

### Mixed effects model development

We implemented a linear mixed-effects model using the *lmer* function from the lmerTest© package. This approach appropriately accounted for the hierarchical structure of the data, with multiple observations nested within the patients. The initial model included the following predictors: Age, Height, Weight, Polypharmacy, Average Volume of the Superficial Masseter Muscle (average of left and right superficial masseter muscles), Average Volume of the Deep Masseter muscle (average of left and right deep masseter muscles), Bite Force Region, Maxilla Tooth Mobility Degree [29], Mandible Tooth Mobility Degree [29], Average Cross Sectional Area of the Muscles at C3 Vertebrae Level (average CT scan and MRI scan measurement), Total number of Teeth, and Average Pinching Strength (average of left and right hand). Patient identification (ID) was included as a random effect to account for repeated measurements. A systematic backward elimination procedure was employed to refine the model, removing non-significant predictors sequentially based on likelihood ratio tests. The following variables were eliminated in order following the backward selection procedure: Age (*p* = 0.919), Average Cross Sectional Area of Muscles at C3 Vertebrae Level (*p* = 0.768), Polypharmacy (*p* = 0.470), Average Volume of the Deep Masseter Muscle (*p* = 0.449), Height (*p* = 0.211), Average Pinching Strength (*p* = 0.188) and Total Number of Teeth (*p* = 0.084).

### Final prediction model results

The final mixed-effects model for bite force prediction included six fixed factors: Weight, Average Volume of the Superficial Masseter Muscle, Bite Force Region, Maxilla Tooth Mobility Degree and Mandible Tooth Mobility Degree. The model is shown in Table 2.

**Table 2:** Summary of the fixed effects from the final mixed-effects model for oncology patient bite force prediction. The resulting linear formula is: bite force = 109.98–1.34 * Weight + 11.47 * Average volume of the superficial masseter muscle − 41.36 * bite force region (Premolar region) − 54.99 * bite force region (Cuspid/Incisor region)- 45.06 * Mobility degree 1 maxilla tooth − 11.55 * Mobility degree 1 mandible tooth − 78.76 * Mobility degree 2 mandible tooth

Parameter	aβ	95% Confidence Interval (CI)	*p*-value
Intercept	109.98	[36.51, 183.73]	0.004
Weight (kg)	−1.34	[−2.50, −0.17]	0.026
Average Superficial Masseter Muscle Volume (cm^3^)	11.47	[6.47, 16.45]	< 0.001
Bite Force Region (Molar Region)	Ref.		
Bite Force Region (Premolar Region)	−41.36	[−55.63, −27.12]	< 0.001
Bite Force Region (Cuspid/Incisor Region)	−54.99	[−91.58, −18.38]	0.003
Maxillary teeth no mobility	Ref.		
Maxillary teeth mobility 1	−45.06	[−85.50, −5.04]	0.028
Mandibular teeth no mobility	Ref.		
Mandibular teeth mobility 1	−11.55	[−44.26, 21.23]	0.486
Mandibular teeth mobility 2	−78.76	[−129.08, −28.62]	0.002

Weight demonstrated a significant negative association with bite force (aβ = −1.34 N/kg, 95% CI = [−2.50, −0.17], *p* = 0.004), indicating that higher body weight is associated with lower bite force. Conversely, the higher Average Volume of the Superficial Masseter Muscle was significantly associated with higher bite force (aβ = 11.47 N/cm^3^, 95% CI = [6.47, 16.45], *p* < 0.001). The region where bite force was measured (Bite Force Region) significantly influenced the outcomes. Compared to the Molar Region (reference), the Premolar Region showed substantially lower bite force (aβ = −41.36 N, 95% CI = [−55.63, −27.12], *p* < 0.001), and the Cuspid/Incisor Region demonstrated an even greater reduction in force (aβ = −54.99 N, 95% CI = [−91.58, −18.38], *p* = 0.003). Higher degrees of teeth mobility in both the maxilla and mandible impacted bite force negatively. Specifically, a mobility degree 1 of the maxillary teeth was associated with reduced bite force compared to the axillary teeth without mobility (reference) (aβ = −45.06 N, 95% CI =[−83.50, −5.04], *p* = 0.028). Increased mandibular teeth mobility issues (degree 1) showed a non-significant association with reduced bite force (aβ = −11.55 N, 95% CI = [−44.26, 21.23], *p* = 0.486), compared to the reference category (Mandibular teeth no mobility). Visually increased mandibular teeth mobility problems (Mandible Tooth Mobility Degree 2) had an even more pronounced negative effect (aβ = −78.76 N, 95% CI = [−129.08, −28.62], *p* = 0.002).

### Model performance

The final model demonstrates explanatory performance with a conditional R² of 0.777. A 10-fold cross-validation gave an average MAE of 40.7 N, with fold-specific errors ranging from 23.5 to 64.6 N (SD ± 13.9). A post-hoc power analysis was conducted to assess the adequacy of the sample size for detecting the observed effects. Most of the predictors showed sufficient statistical power: Mobility Degree Mandible Tooth (82.8%, 95% CI [80.3, 88.1]), Average Volume of the Superficial Masseter Muscle (99.5%, 95% CI [98.8, 99.8]), and Bite Force Region (100.0%, 95% CI [99.6, 100.0]). The power of two predictors was below the conventional 80% threshold: Mobility Degree Maxilla Tooth (65.6%, 95% CI [62.6, 68.5]) and Weight (60.4%, 95% CI [57.3, 63.5]). Despite the lower power of these variables (i.e., a higher chance of false negative findings), significant effects were detected (*p* = 0.028 and *p* = 0.004, respectively), indicating the robustness of these associations, even with the current sample size.

## Discussion

The insight into the factors contributing to maximum voluntary bite force in head and neck cancer patients enabled the construction of a predictive model that can be applied to patient-specific cases (see Table 2). By developing a comprehensive predictive model with high explanatory performance, we addressed the limitations in the existing approaches that often rely on limited sets of predictors, or predictors that are very complex to derive [34, 35]. Our model demonstrated strong explanatory performance (conditional R² = 0.777), emphasizing the importance of the selection of relevant anatomical, physiological, and clinical factors in bite force estimation. The conditional R^2^ aligns closely with high-performance biomedical models [36], while maintaining clinical usability, which makes it exceptionally strong for biomechanical studies in human populations [37]. While high performance computer modeling approaches, like Aftabi et al. [38] for postoperative bite-forces, may yield similar results, our model is significantly easier to apply in clinical practice. We expect to use this model to predict the maximum bite force of head and neck oncology patients that require maxillofacial reconstruction, involving loading forces from the masticatory system, without actually having to measure the bite forces. Measuring head and neck oncology patients’ maximum bite force is not always feasible. Using a daily calibrated sensor is time consuming and impractical, and requires dedicated technical personnel. Tumor location, pain and emotional distress may all limit measurement device placement and patient compliance [4, 39].

Commercially available bite force sensors were considered to be of limited applicability for this study. Commercially available sensors are generally designed to span the entire dental arch, which does not allow for point force measurement [40, 41]. Point-based maximum biteforce measurements provide a detailed insight in the maximum bite force at multiple locations in the mouth (for instance incisor or molar region), which was a requirement to develop the predictive model. In literature differences among bite force sensors are described and some applicable sensors are mentioned, but are not commercially available [5, 42]. These designs were taken into consideration when designing the sensor for this study. Using bite force sensors to directly measure bite force would not work for patients experiencing pain during biting. Therefore, our accurate predictive model is a necessity for 3D modelling, finite element analyses, and 3D design of patient-specific mandibular reconstruction plates.

The positive association between superficial masseter muscle volume and bite force aligns with prior research [43, 44]. In the diagnostic work-up of patients suffering from head and neck cancer, MRI is the most routine diagnostic modality. The assessment of masseter muscle volumes can be routinely included in this, thus providing information with no added costs, and minimal effort. This makes the outcome of our study directly applicable to further clinical studies, e.g., in optimizing the design of mandibular reconstruction plates [9]. Segmentation of the muscle volumes was performed by a deep learning segmentation model that has been validated [28] and, thus, inter-observer discrepancies are excluded. The importance of the superficial masseter muscle over the other masticatory muscles has been described before [44, 45]. The measurement-region influences bite force outcomes, with posterior regions exhibiting higher forces than anterior regions. This observation is consistent with biomechanical advantages offered by the posterior teeth, due to their anatomical positioning and occlusal surface area, and concurs with earlier findings [46, 47]. Additionally, maxilla and mandibular teeth mobility emerged as a key factor that affects bite force negatively. This aligns with previous studies [48, 49], and highlights the impact of dental stability on masticatory performance. The issue with teeth mobility affecting the bite force has, potentially, a temporary effect on the bite force since it might be possible to treat this [50, 51].

Contrary to previous studies, body weight was found to have a significant negative moderate association with bite force [52, 53]. This discrepancy may reflect differences in patient populations; high body weight in oncology patients could indicate poorer overall health or muscle quality rather than increased strength. People with a high body mass index (BMI) may still be undernourished, which can be reflected in muscle strength [54–56]. Nutritional insufficiencies, as well as being overweight or obese, can affect muscle strength negatively. Head and neck cancer patients with medium to high malnutrition risk are shown to be more likely to be frail, compared to patients with a low malnutrition risk [57]. Surprisingly, the cross-sectional muscle area at the C3 level, a sarcopenia measure [58] used on head and neck oncology patients [59], did not contribute to the performance of the model. Since there is a correlation between the cross-sectional muscle area at the C3 level and masseter volume [60], it is likely that the explanatory effect of the superficial masseter muscle is larger than that of the cross-sectional muscle area at the C3 level.

Despite these strengths, limitations should be acknowledged. Although the majority of the predictors in our model demonstrate sufficient statistical power (≥ 80%) to detect their effects, two predictors—maxilla teeth mobility and weight—had power values below this threshold (65.6% and 60.5%, respectively). These lower power levels indicate a higher chance of type II errors (i.e., false negative findings). However, it is important to note that both predictors were statistically significant in our analysis (i.e., a positive finding), suggesting that their associations with bite force are evident despite the limited power. The use of multiple imputation to handle missing data, variance inflation factor analysis to address multicollinearity, and backward selection for model refinement, ensured that only meaningful predictors were included in the final model. Furthermore, the hierarchical structure of the linear mixed-effects model accounted for within patient repeated measurements, enhancing the reliability of our findings.

Regarding clinical use, this model offers a robust tool for personalized treatment planning and implant design. By predicting bite forces accurately based on patient-specific factors, it enables tailored approaches that could mitigate complications such as stress shielding in mandibular reconstructions [9]. The preoperatively predicted bite force should be considered as a ‘worst’-case loading scenario, for the postoperative situation, which the mandibular reconstruction plate should be able to withstand while not adding unnecessary stiffness to the implant and thus reducing stress-shielding. By iteratively designing and simulation the mandibular reconstruction plate design, the step from ‘shape-specific’ to truly ‘patient-specific’ can be made, optimizing both mechanical performance and biological integration. When clinically using the model, in some unique cases implausible outputs (e.g. negative bite forces predictions for patients with high weight and mobility values) are theoretically possible, the user should place the outputs in clinical context. Future research should focus on external validation of the current model. Additionally, advancements in metamaterial-based reconstruction plates designed to accommodate individual bite forces present an exciting avenue for reducing stress shielding while maintaining structural integrity.

## Conclusion

This study represents a significant step forward in understanding and predicting oral and maxillofacial surgery patients’ bite forces. The final model, which explains 77.7% of bite force variance, highlights the importance of superficial masseter muscle volume, body weight, bite force measurement region, and dental mobility. Clinically, this model supports personalized mandibular reconstruction plate design, enabling optimization of implant strength to balance durability and avoid stress shielding.

## Data Availability

The data supporting the findings of this study are not publicly available due to privacy and ethical restrictions. The dataset contains patient information that cannot be sufficiently anonymized to ensure participant confidentiality, in accordance with institutional policies and applicable data protection regulations. Therefore, the data cannot be accessed openly.
